# Computational Fluid Dynamic Optimization of Micropatterned Surfaces: Towards Biofunctionalization of Artificial Organs

**DOI:** 10.3390/bioengineering11111092

**Published:** 2024-10-30

**Authors:** Wenxuan He, Aminat M. Ibrahim, Abhishek Karmakar, Shivani Tuli, Jonathan T. Butcher, James F. Antaki

**Affiliations:** 1 Sibley School of Mechanical and Aerospace Engineering, Cornell University, Ithaca, NY 14853, USA; wh444@cornell.edu; 2 Meinig School of Biomedical Engineering, Cornell University, Ithaca, NY 14853, USA; ami26@cornell.edu (A.M.I.); jtb47@cornell.edu (J.T.B.)

**Keywords:** LVAD, mechanical valves, thrombosis, endothelial cells, hemocompatibility, mechanical devices

## Abstract

Modifying surface topography to prevent surface-induced thrombosis in cardiovascular implants allows endothelialization, which is the natural thrombo-resistance of blood-contacting surfaces, and is deemed to be the only long-term solution for hemocompatible materials. We adapted a simulation framework to predict platelet deposition on a modified surface and developed an optimization strategy to promote endothelial retention and limit platelet deposition. Under supraphysiological bulk shear stress, a maximum of 79% linear coverage was achieved. This study concludes that the addition of microtrenches promotes endothelial retention and can be improved through the optimal selection of geometric parameters.

## 1. Introduction

Artificial biomaterials which are in contact with blood are widely used in implantable cardiovascular devices, including heart valves and left ventricular assist devices (LVADs). A significant challenge in the application of artificial biomaterials is surface-induced thrombosis, which results from protein adsorption and platelet adhesion and activation [1,2]. Therefore, long-term coagulation management is required. However, this comes with its own risks, primarily an increased chance of hemorrhage [3]. Research indicates that patients with mechanical prosthetic heart valves face a significant risk of bleeding or clotting, with an accumulated risk as high as six percent per patient-year [4]. A report on patients with advanced heart failure who were treated with the HeartMate 3 LVAD presented major bleeding events of 0.61 per patient-year, the greatest among all major adverse events [5].

This phenomenon starts with circulating plasma protein absorption on blood-contacting artificial material surfaces—this process includes smaller proteins like human serum albumin, followed by larger proteins such as fibrinogen [6]. The adsorbed fibrinogen provides binding sites for platelets, which then become activated and release factors that promote further platelet aggregation and coagulation [7]. The foreign surface can also activate the intrinsic coagulation pathway through contact activation of Factor XII, leading to thrombin generation and fibrin formation [6,7]. This combination of activated platelets and the coagulation cascade results in the formation of thrombus on the blood-wetted surfaces.

In contradistinction, endothelial cells (ECs) provide the natural thrombo-resistant lining of the blood-contacting surface and are deemed to be the only long-term hemocompatible material [8,9]. Healthy ECs utilize pathways that include the ecto-ADPase/CD39/NTPDase pathway, which limits the propagation of platelet activation and reduces the risk of thrombus formation; and the PGI2 and Nitric Oxide (NO) pathways, which inhibit platelet activation and aggregation through the stimulation of cAMP and cGMP production, respectively [10].

Therefore, there is a clear benefit for the endothelialization of cardiovascular implants [6,11]. One method of promoting endothelialization is through surface modification processes [12]. For instance, surfaces covered with microspheres (aka. sintering) are currently used in left ventricular assist devices (LVADs) [13]. Such sintered surface topography is adopted in the hope of growing neointima tissue and a continuous endothelium lining to shield the artificial surface from direct contact with blood, ultimately reducing surface thrombogenicity [13,14]. Unfortunately, studies published over the decades indicate that surface sintering can lead to unpredictable results; hence, it is not a reliable method to avoid thromboembolism [13]. A critical limiting factor is supraphysiological wall shear stress (WSS), which commonly occurs in medical devices [15,16]. This limits endothelial attachment and causes embolization. For example, the typical WSS associated with mechanical valve leaflets can range from 250 to 750 dynes/cm^2^ [17,18], far in excess of the normal WSS in blood vessels (less than 50 dynes/cm^2^) which allows the endothelium to maintain its monolayer structure and perform its anticoagulation function [16].

Previous studies have shown that creating groove-like surface topography, named “microtrenches,” can enhance EC retention in a supraphysiological shear environment, and consequently reduce platelet adhesion [17,19,20,21,22,23,24]. The underlying mechanism is that trenches create one or more vortices that attenuate WSS to a level tolerable for ECs. Daxini et al. created a grooving pattern of 32 μm deep and 35°, lowering the WSS by 23%, which helped to promote EC wound recovery [22]. To work with the shear range above 500 dynes/cm^2^, Frendl et al. created several fold deeper trenches in pyrolytic carbon [23]. Therefore, the modified surface encouraged EC retention, inducing the release of anticoagulant molecules (e.g., nitric oxide) and reducing platelet adhesion greatly [23]. The efficacy of “microtrenches” was demonstrated to provide EC protection for over 48 h of perfusion [23]. One of the objectives of the present study is to perform numerical simulations of these experimental results and further optimize the dimensions of the microtrench to maximize EC coverage. This is achieved by coupling the automatic optimization software CAESES^®^ (CAESES 5.2 on Linux, Friendship Systems AG, Potsdam, Germany) with the open-source CFD toolkit OpenFOAM.

## 2. Materials and Methods

### Flow Field Simulation and the Optimization of Microtrenches

Numerical optimization of blood flow over a biomaterial surface populated with a series of microtrenches was performed. The computational domain consisted of a parallel plate channel with a height of 0.02” (~0.508 mm). The primary geometric variables of the trenches were as follows: the height (h), width (w), and draft angle (α) of the walls, and the gaps (d) therebetween. For all simulations, a constant velocity was specified at the inlet corresponding to the experimental procedure. The blood flow was modeled as a homogeneous Newtonian fluid governed by the Navier–Stokes equations. The corresponding Reynolds number was 83.2; therefore, the flow was assumed to be laminar. The viscosity and density of the blood were specified as 3.5 cP and 1050 kg m^−3^, respectively. A previously validated multi-constituent thrombosis model [25] was applied to simulate the anticoagulant effect of the endothelial cell (EC)-covered surface. The presence of the endothelial layer was modeled by altering the reaction rate that controls the flux of activated platelets to the surface.

We conducted a fully automatic optimization using parametric CFD optimization of the design variables. The geometric variables were provided by CAESES coupled with OpenFOAM. The objective function f was the dimensionless fraction of the projected area, having a WSS within the range of 10–50 dynes/cm^2^.

(1) $$f=\oint (A_{WSS}\cdot \vec{n})/\oint (A_{trench}\cdot \vec{n})\;$$

(2) $$A_{WSS}=\left\{\begin{array}{c} A_{trench},\;\\ 0, \end{array}\begin{array}{c} \;\;if\;10<\tau <50\\ otherwise \end{array}\right.$$

where $\vec{n}$ is the normal vector of the surface, $A_{trench}$ is the incremental surface area, $\oint (A_{trench}\cdot \vec{n})$ is the projected cumulative area, and $A_{WSS}$ is the surface area where the wall shear stress τ (dynes/cm^2^) is within the threshold.

These thresholds were chosen based on physiological WSS values and in vitro results proven to maintain EC monolayer integrity [16]. Additional constraints were applied, as summarized in Table 1. The matrix of the combined effect of these three parameters is illustrated in Figure S1. Each parameter could pick one of three values, which yielded 27 designs in total. The index H1A1W1 refers to the smallest height, angle, and width, respectively.

## 3. Results

### 3.1. Simulation of Platelet Adhesion

Initial simulations were conducted to mimic the published in-vitro experimental results of Frendl et al. [23]. Human endothelial cells seeded within collagen-coated microtrenches were exposed to a bulk shear stress of 600 dynes/cm^2^ and human platelets afterward. The dimensions of the microtrenches were 700 μm deep and 400 μm wide.

**Figure 1:**
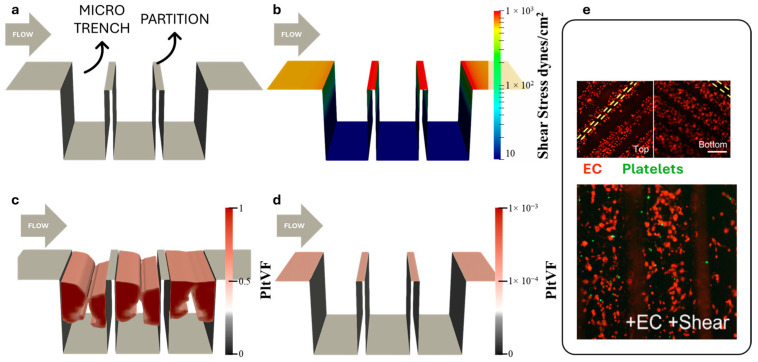
CFD and thrombosis simulation of vertical microtrench patterned surface. (**a**) Simulation domain. (**b**) Wall shear stress. (**c**) Simulation of thrombus within collagen-coated microtrenches at t = 3001. (**d**) No thrombus formation on EC-coated microtrenches. (**e**) Confluent EC (red) are retained after 48 h of 600 dynes/cm^2^ steady bulk flow and images of platelet (green) adhesion to the EC-coated microtrench surface. (Yellow dash lines indicate partition.) [23].

The simulation employed a 2D domain corresponding to a channel with a fixed height, with the bottom surface featuring three trenches (Figure 1a). The domain included sections of active collagen-coated surfaces and inactive surfaces pre-seeded with ECs. At the inlet, a uniform velocity was applied to achieve a wall shear stress of 600 dynes/cm^2^ within the entrance length (and upper surface.) Figure 1b shows that the wall shear stress distribution within the microtrench region was reduced by two orders of magnitude. The reduction in WSS was most dramatic within the microtrenches but the shear stress along the top of the partitions elevated the bulk WSS by approximately 60%.

The results of the multi-constituent thrombosis model are provided in Figure 1c, revealing that collagen-coated microtrenches yield noticeable thrombus formation inside the microtrenches at the time point of 50 min. The assumed 100% EC-coated microtrenches (Figure 1d), in contrast, were confirmed to limit platelet adhesion—corresponding to the experimental results (Frendl et al.), Figure 1e demonstrates confluent ECs (red dots) retained on the microtrench surface after 48 h of perfusion and the absence of platelets (green dots) on microtrenches surfaces.

### 3.2. Optimization of Microtrenches

**Figure 2:**
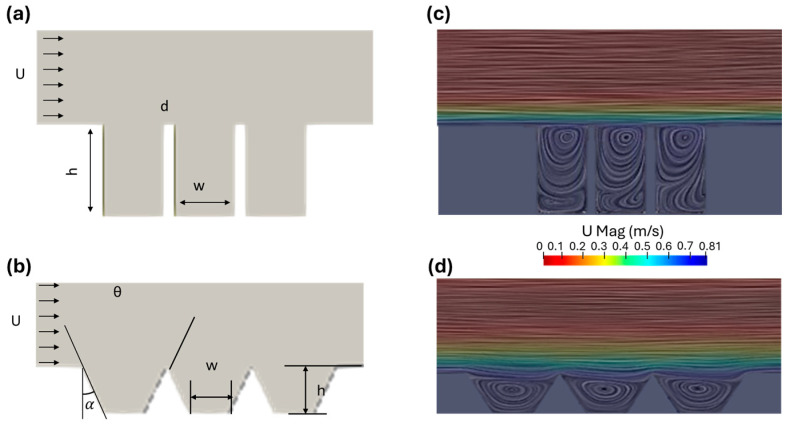
An overview of the geometry parameters and flow dynamics around different trench geometries. (**a**) A visualization of the vertical trench geometry. A constant velocity was specified at the inlet from left to right. The height (h), width (w), and the gaps (d) are free variables. (**b**) A visualization of the trapezoidal trench geometry. A constant velocity was specified at the inlet from left to right. the height (h), width (w), and draft angle (α) of the walls are free variables. (**c**) Streamlines around the vertical trench, color-coded by the velocity magnitude. (**d**) Streamlines around the trapezoidal trench, color-coded by the velocity magnitude.

Figure 2a provides a schematic of the initial geometry, corresponding to Friendl et al. [23], and Figure 2b illustrates a generalized geometry in which the height (h), angle (α), and width (w) are free variables, and the wall thickness is allowed to vanish to zero. Typical streamlines within the trenches, as shown in Figure 2c,d, reveal the presence of vortex formations.

### 3.3. Optimization of Trapezoidal Trench Geometry

**Figure 3:**
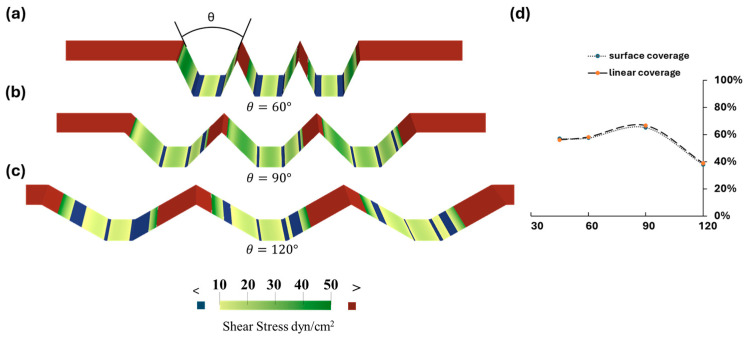
(**a**–**c**) An evaluation of the WSS of trapezoidal trenches with varying angles from 60°, 90°, and 120°. The color-coding indicates areas with WSS values: green represents a WSS within the threshold range of 10–50 dynes/cm^2^, red denotes areas above 50 dynes/cm^2^, and blue represents areas below 10 dynes/cm^2^. (**d**) The surface coverage of the optimal WSS regions, along with the projected area coverage for comparative analysis across the designs.

The optimization of microtrenches geometries is based on the evaluation of the coverage of the area with a WSS ranging from 10 dynes/cm^2^ to 50 dynes/cm^2^. It can be seen in Figure 3a–c that the WSS at the bottom corners of the microtrenches is less than 10 dynes/cm^2^, as seen previously in Figure 1b the bottom surface, and hence is not optimal for maintaining EC monolayer integrity.

A pilot study in which the angle θ varied from 60°, 90°, and 120° revealed a drastic variance in the profile of areas with a desirable WSS level, indicating that the angle has a significant impact on WSS distribution and hence limits undesirable WSS bands. A quantitative comparison of coverage with a fixed height of 150 μm and width of 110 μm revealed that the right-angle microtrenches exhibited the most favorable WSS distribution compared to 60° and 120° configurations. See Figure 3d.

**Figure 4:**
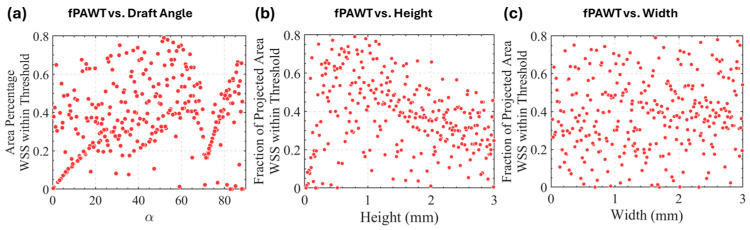
An exploration of design parameters and their influence on the projected area coverage. (**a**) Angle. (**b**) Height. (**c**) Width.

An auto-optimization was conducted to identify the optimal combination of design parameters—draft angle (α), height (h), and width (w)—to maximize the objective function. Figure 4 shows their effect on the objective function. The optimal configuration consisted of a trench height of 0.79 mm, a width of 1.6 mm, and a draft angle of 51.8°. However, as shown in Figure 4c, it is interesting to note that the width of the trench does not exhibit a direct correlation with the objective function, where no optimized value was found in this search.

## 4. Discussion

This study presented a framework for optimizing surface topography to promote endothelial cell retention and improve the biocompatibility of implantable medical devices. Specifically, this study sought to optimize a trench-shaped surface topography under a single ultra-high shear environment, representative of the condition in devices such as mechanical prosthetic heart valves and inflow cannula of ventricular assist devices.

The optimization process began with pre-validated vertical microtrenches, investigating how variations in the trench dimensions (height, width, and gaps) could influence the WSS profile and subsequently influence EC coverage. However, the EC coverage achieved with this design was limited. The rationale for transitioning from vertical to trapezoidal trenches was twofold. Firstly, we observed that decreasing the gap between the vertical trenches did not result in a significant alteration of the WSS distribution inside the trench, as shown in Figure S2, yet the partition between two trenches is guaranteed to have undesirable high shear stress. This finding motivated us to reduce the gap further to increase EC coverage while maintaining the mechanical integrity of the topography. Secondly, although the WSS on the vertical trench was optimal, it failed to increase the projected EC coverage on a limited area of surface.

It is worth noting that a few assumptions were made; thence, key simplifications have been applied for computing at a reasonable expense yet achieving realistic results. First, we assumed a 2D parallel plate channel which was chosen to be consistent with the in vitro validation experiment. Second, a uniform inlet velocity was introduced, and an ample entrance length allowed for the flow field to become fully developed at the leading edge of the trench. Third, we applied a steady-state flow instead of the pulsatile blood flow, which does not account for the complexity of the flow in vivo. This simulation also does not account for biological responses beyond the initial interaction of platelet deposition. The behavior of endothelial cells and thrombosis formation could vary under different flow conditions, including endothelial cell migration, mitosis, and/or the secretion of anti-thrombotic substances in response to the shear stress.

Future studies should address these limitations by incorporating the cellular interaction between the endothelium and the blood, the secretion and transport of the anti-thrombotic substances, and the effect on platelet activation and aggregation. Such a model could also be applied in complex flow conditions, e.g., pulsatile flow, to represent the physiological environment and more complex geometries.

The width of the trenches was examined as a parameter that could not be optimized within the constraints of this study. It was illustrated through an extreme case, where the microtrenches are reduced to a parallel plate. See Figure S3. Ideally, the wall shear stress is governed by the total height of the flow, as explained in Equation (3):

(3) $$\tau \;\propto \;\frac{1}{h_{total}}$$

where τ represents the wall shear stress in dynes/cm^2^, and $h_{total}$ is the total height of the channel without trenches in mm. We hypothesize that there exists a critical height of the channel (10.952 mm), such that the WSS equals the desired threshold (10 dynes/cm^2^). To validate this mathematical interpretation, we set the height at the critical value and compared the projected area coverage with varying channel widths. The results show that the objective function approaches an asymptotic value, while the w extends to infinity. See Figure S3. This might result in an optimized design that has a width at the upper bound, however such a value lacks practical meaning. In reality, the width of the trench is constrained by manufacturing limitations, mechanical stability, and other factors. Though it is not directly optimized, its selection is still guided by practical applications. Nevertheless, the addition of microtrenches will promote endothelial retention and can be improved through the optimal selection of geometric parameters.

**Figure 5:**
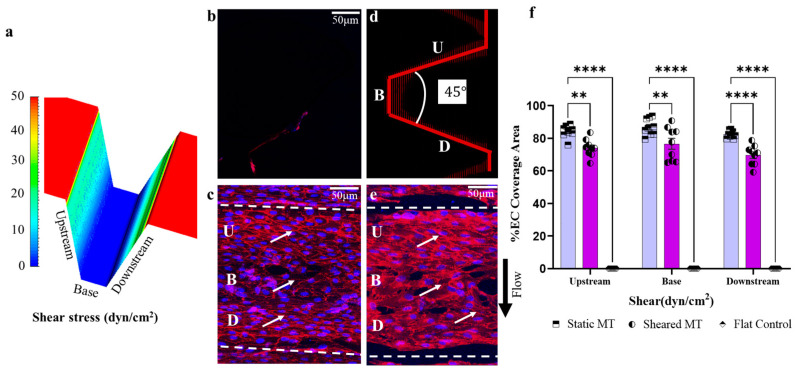
Endothelial retention under high shear stress. (**a**) A CFD simulation of the trapezoidal microtrench (MT) at a high pump flow rate. Immunohistochemistry images of the MT showing seeded endothelial cells: the flat MT control (**b**), the static MT with no flow (**c**), a 3D projection of the sheared MT (**d**), and the sheared MT at 48 h post high shear stress (**e**). The arrows show the mature endothelial junction, blue (DAPI), red (VE Cadherin). U, B, and D correspond to the Upstream, Base, and Downstream regions; (**f**) quantification of EC coverage at the different regions. Data represent the mean ± SEM. n represents the number of independent biological samples with triplicates measured per sample. Two-way ANOVA with Tukey’s HSD test. **** *p* = *p* < 0.0001; ** *p* = *p* < 0.005.

As a proof of concept of our optimized geometry, we evaluated endothelial retention on a trapezoidal microtrench by embossing a 45° trapezoidal geometry (shown using a fluorescence image in Figure 5d) on a polymeric substrate. We then coated the embossed channel with collagen and seeded the channels with endothelial cells. CFD simulation, as seen in Figure 5a, determined that the ECs experienced a shear stress range of about 5-fold less than the applied bulk shear stress (120 dynes/cm^2^), which supports the throttling capacity of the trapezoidal microtrench. Also, we found that the trench surface provided three times the surface area (Upstream, Base, and Downstream) in comparison to the flat control, effectively increasing the surface area for EC adhesion. Twenty-four hours post-seeding, we applied continuous shear stress at a maximum pump flow rate for 48 h. Endothelial retention on the flat microtrench control was completely diminished, as shown in Figure 5b. However, we found a retained, confluent EC monolayer with visible junction integrity in the Upstream (U), Base (B), and Downstream (D) regions of the no-flow control microtrench (Figure 5c) and the sheared sample microtrench (Figure 5e). Immunofluorescence staining showed an expressed endothelial cell junction, as marked by VE Cadherin staining (CD144 monoclonal antibody, Bio-Rad, United States). Although comparable, we observed significant differences in the endothelial coverage area fraction between the different regions of the sheared microtrench (84%, 87%, and 82%) vs. the control (74%, 77%, and 69%), as shown in Figure 5f.

The above results validate endothelial retention in the optimized trapezoidal microtrench under high shear stress, as ECs remained adhered to the microtrench channel, similar to Frendl et al. [23]. The downstream region in the sheared samples showed higher degradation, which can be attributed to the force of fluid against the downstream wall. Furthermore, the endothelial retention rate corroborated our CFD optimization, as we observed an above 50% retention rate at the different regions within the microtrench post-shear stress, thus alluding to its potential to support more EC segments for monolayer formation. Although the height, width, and angle used in this experiment varied from the optimized geometry and a cell culture medium was adopted to assess the long-term retention of the ECs, the results of EC retention on such microtrenches were encouraging regarding the feasibility of seeding and the protection of the EC monolayer. In the future, we plan to address the difference between the simulation and the experiment and examine the anti-platelet effect of the EC-coated microtrenches by incorporating blood as a fluid medium.

## Figures and Tables

**Table 1:** Design space for trapezoidal microtrenches.

Design Variables	Lower Bound	Upper Bound	Average Interval
Height (mm)	0.01	3	0.1
Width (mm)	0.01	3	0.1
Angle * (°)	0	90	0.3

* Angle θ here is twice the draft angle α.

## Data Availability

The original data presented in this study are available from the corresponding author, Dr. James F Antaki [antaki@cornell.edu].

## Supplementary Materials

The following supporting information can be downloaded at https://www.mdpi.com/article/10.3390/bioengineering11111092/s1, Figure S1: Visualization of 27 design configurations based on combinations of three design parameters: height (H), draft angle (A), and width (W). Figure S2: Comparison of shear stress distribution on the surface of two trenches with different gap widths (d). Figure S3: Exploration of an extreme design case.

## References

[1] Gorbet M.B., Sefton M.V. (2004). Biomaterial-Associated Thrombosis: Roles of Coagulation Factors, Complement, Platelets and Leukocytes. The Biomaterials: Silver Jubilee Compendium.

[2] Labarrere C.A., Dabiri A.E., Kassab G.S. (2020). Thrombogenic and Inflammatory Reactions to Biomaterials in Medical Devices. Front. Bioeng. Biotechnol..

[3] Aparicio H.J., Benjamin E.J., Callaway C.W., Carson A.P., Cheng S., Elkind M.S.V., Evenson K.R., Ferguson J.F., Knutson K.L., Lee C.D. (2021). Heart Disease and Stroke Statistics-2021 Update A Report from the American Heart Association. Circulation.

[4] Cannegieter S.C., Rosendaal F.R., Briët E. (1994). Thromboembolic and Bleeding Complications in Patients with Mechanical Heart Valve Prostheses. Circulation.

[5] Mehra M.R., Uriel N., Naka Y., Cleveland J.C., Yuzefpolskaya M., Salerno C.T., Walsh M.N., Milano C.A., Patel C.B., Hutchins S.W. (2019). A Fully Magnetically Levitated Left Ventricular Assist Device—Final Report. N. Engl. J. Med..

[6] Kuchinka J., Willems C., Telyshev D.V., Groth T. (2021). Control of Blood Coagulation by Hemocompatible Material Surfaces—A Review. Bioengineering.

[7] Vogler E.A., Siedlecki C.A. (2009). Contact Activation of Blood-Plasma Coagulation. Biomaterials.

[8] Furukawa K.S., Ushida T., Sugano H., Tamaki T., Ohshima N., Tateishi T. (2000). Effect of Shear Stress on Platelet Adhesion to Expanded Polytetrafluoroethylene, a Silicone Sheet, and an Endothelial Cell Monolayer. ASAIO J..

[9] Ahmann K.A., Johnson S.L., Hebbel R.P., Tranquillo R.T. (2011). Shear Stress Responses of Adult Blood Outgrowth Endothelial Cells Seeded on Bioartificial Tissue. Tissue Eng.-Part A.

[10] Jin R.C., Voetsch B., Loscalzo J. (2005). Endogenous Mechanisms of Inhibition of Platelet Function. Microcirculation.

[11] Dangas G.D., Weitz J.I., Giustino G., Makkar R., Mehran R. (2016). Prostheticurrentc Heart Valve Thrombosis. J. Am. Coll. Cardiol..

[12] Wolfe J.T., Shradhanjali A., Tefft B.J. (2022). Strategies for Improving Endothelial Cell Adhesion to Blood-Contacting Medical Devices. Tissue Eng. Part B Rev..

[13] He W., Butcher J.T., Rowlands G.W., Antaki J.F. (2023). Biological Response to Sintered Titanium in Left Ventricular Assist Devices: Pseudoneointima, Neointima, and Pannus. ASAIO J..

[14] Zapanta C.M., Griffith J.W., Hess G.D., Doxtater B.J., Khalapyan T., Pae W.E., Rosenberg G. (2006). Microtextured Materials for Circulatory Support Devices: Preliminary Studies. ASAIO J..

[15] Miyamoto T., Nishinaka T., Mizuno T., Tatsumi E., Yamazaki K. (2015). LVAD Inflow Cannula Covered with a Titanium Mesh Induces Neointimal Tissue with Neovessels. Int. J. Artif. Organs.

[16] Robotti F., Franco D., Bänninger L., Wyler J., Starck C.T., Falk V., Poulikakos D., Ferrari A. (2014). The Influence of Surface Micro-Structure on Endothelialization under Supraphysiological Wall Shear Stress. Biomaterials.

[17] Gong X., Yao J., He H., Zhao X., Liu X., Zhao F., Sun Y., Fan Y. (2017). Combination of Flow and Micropattern Alignment Affecting Flow-Resistant Endothelial Cell Adhesion. J. Mech. Behav. Biomed. Mater..

[18] Yoganathan A.P., Chaux A., Gray R.J., Woo Y.R., DeRobertis M., Williams F.P., Matloff J.M. (1984). Bileaflet, Tilting Disc and Porcine Aortic Valve Substitutes: In Vitro Hydrodynamic Characteristics. J. Am. Coll. Cardiol..

[19] Franco D., Milde F., Klingauf M., Orsenigo F., Dejana E., Poulikakos D., Cecchini M., Koumoutsakos P., Ferrari A., Kurtcuoglu V. (2013). Biomaterials Accelerated Endothelial Wound Healing on Microstructured Substrates under Fl Ow. Biomaterials.

[20] Stefopoulos G., Robotti F., Falk V., Poulikakos D., Ferrari A. (2016). Endothelialization of Rationally Microtextured Surfaces with Minimal Cell Seeding Under Flow. Small.

[21] Bachmann B.J., Giampietro C., Bayram A., Stefopoulos G., Michos C., Graeber G., Falk M.V., Poulikakos D., Ferrari A. (2018). Honeycomb-Structured Metasurfaces for the Adaptive Nesting of Endothelial Cells under Hemodynamic Loads. Biomater. Sci..

[22] Daxini S.C., Nichol J.W., Sieminski A.L., Smith G., Gooch K.J., Shastri V.P. (2006). Micropatterned Polymer Surfaces Improve Retention of Endothelial Cells Exposed to Flow-Induced Shear Stress. Biorheology.

[23] Frendl C.M., Tucker S.M., Khan N.A., Esch M.B., Kanduru S., Cao T.M., García A.J., King M.R., Butcher J.T. (2014). Endothelial Retention and Phenotype on Carbonized Cardiovascular Implant Surfaces. Biomaterials.

[24] Ranjan A., Webster T.J. (2009). Increased Endothelial Cell Adhesion and Elongation on Micron-Patterned Nano-Rough Poly(Dimethylsiloxane) Films. Nanotechnology.

[25] Wu W.T., Jamiolkowski M.A., Wagner W.R., Aubry N., Massoudi M., Antaki J.F. (2017). Multi-Constituent Simulation of Thrombus Deposition. Sci. Rep..

