# Supplementary material for: Computational Fluid Dynamic Optimization of Micropatterned Surfaces: Towards Biofunctionalization of Artificial Organs
Source: Bioengineering (Basel). 2024 Oct 30;11(11):1092. doi: 10.3390/bioengineering11111092 (PMC11591438; doi:10.3390/bioengineering11111092)
Supplement: Supplementary file 1 [file bioengineering-11-01092-s001.zip › bioengineering-3263007-supplementary.pdf]

Supplementary Information

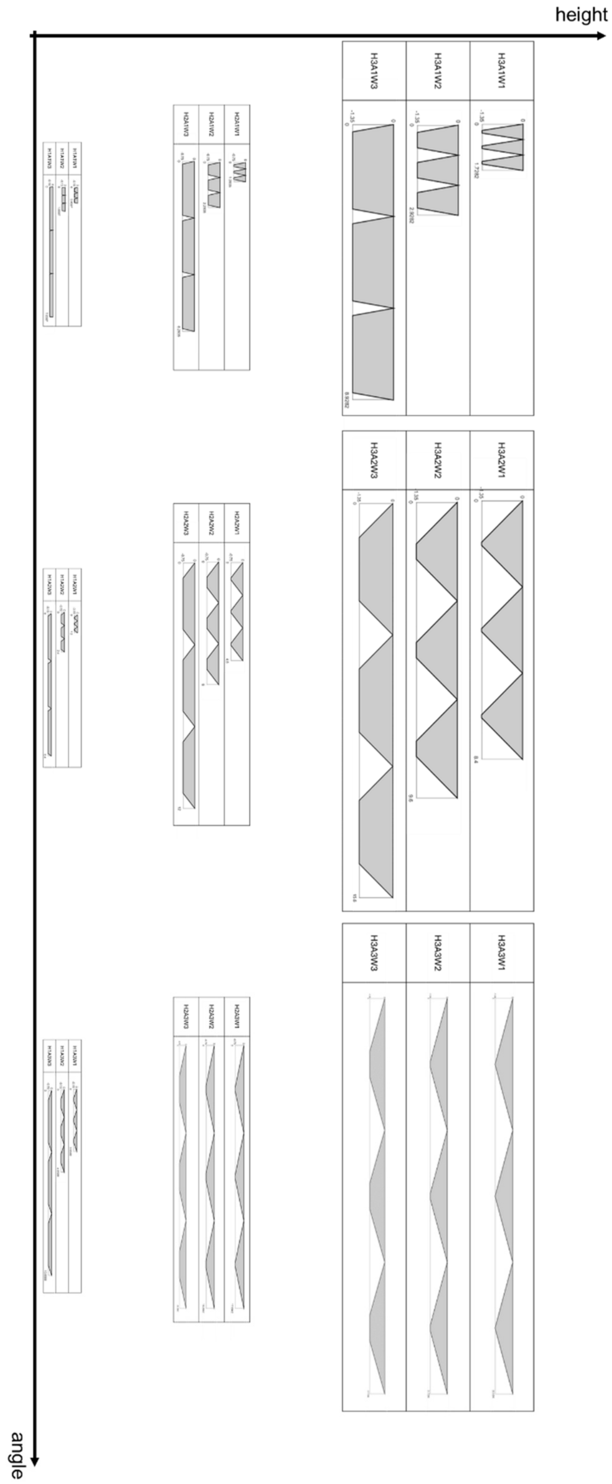

Figure S1. Visualization of 27 design configurations based on combinations of three design parameters: height (H), draft angle (A), and width (W). The designs are labeled according to their parameter values, e.g. "H1A1W1" representing the smallest height, draft angle, and width. Along the x-axis, the draft angle increases from small to large, while the y-axis shows increasing height. Within each sub-box, the width of the trench increases, providing a clear comparison of the design configurations.

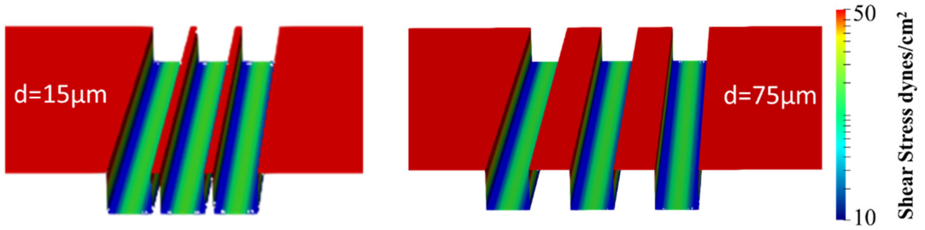

Figure S2 Comparison of shear stress distribution on the surface of two trenches with different gap widths (d). The left and right panels display trenches with a fivefold difference in gap size. Shear stress is represented by color on the trench surface. The minimal difference in shear stress patterns between the two cases indicates that the gap between trenches has a negligible effect on optimizing the shear-covered area.

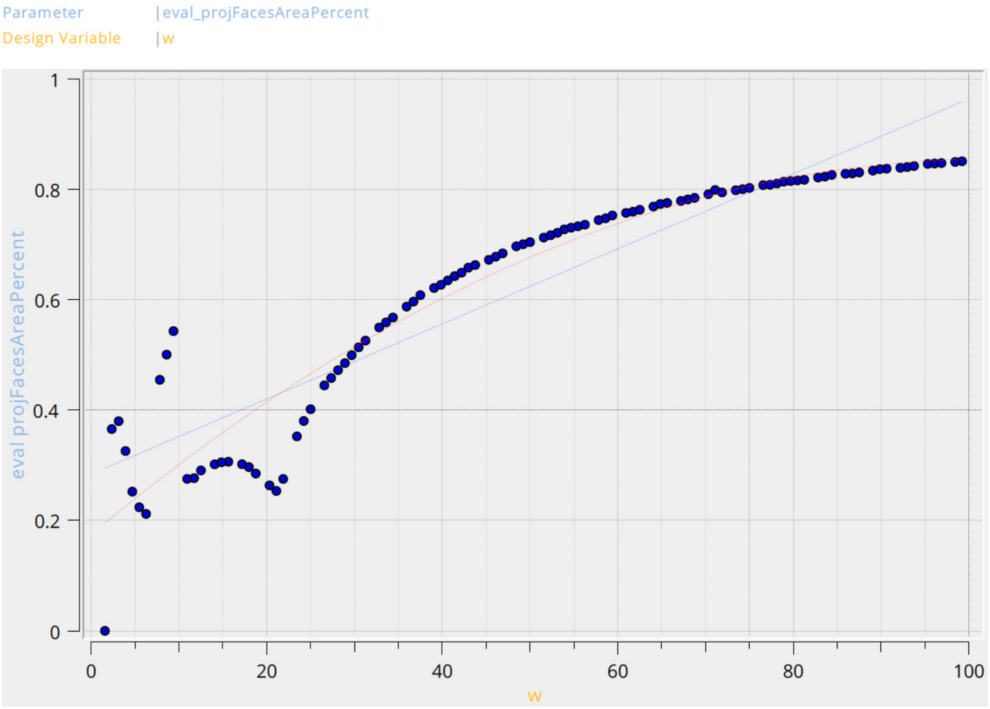

Figure S3. Exploration of an extreme design case. The objective function (projected area coverage) plotted against varying channel widths (w) at fixed height (10.952 mm). This study is used to validate the mathematical interpretation of the extreme case.
